# Case Report: Effective treatment of dystrophic epidermolysis bullosa pruriginosa with tofacitinib

**DOI:** 10.3389/fmed.2025.1648732

**Published:** 2025-10-31

**Authors:** Fang Sun, Zhenzhen Wu, Zhenze Yu

**Affiliations:** Department of Dermatology, Affiliated Aoyang Hospital of Jiangsu University, Zhangjiagang, China

**Keywords:** epidermolysis bullosa, dystrophic epidermolysis bullosa, dystrophic epidermolysis bullosa pruriginosa, JAK inhibitor, tofacitinib

## Abstract

Epidermolysis bullosa (EB) is a heterogeneous group of hereditary skin diseases caused by mutations in structural proteins at the dermal-epidermal junction. Dystrophic epidermolysis bullosa (DEB), one of its main types, is characterized by recurrent pruritic blisters, bullae, atrophy, and scarring, often accompanied by nail dystrophy. Dystrophic epidermolysis bullosa pruriginosa, also known as epidermolysis bullosa pruriginosa (EBP), is a rare clinical subtype of DEB. In addition to the common manifestations of skin blisters and ulcers, patients with EBP also present severe pruritus. Traditional treatments for EBP have limited efficacy. In this study, we report the case of a 59-year-old male patient with EBP who showed significant improvement in skin lesions and pruritus after 10 months of treatment with tofacitinib, a pan-JAK inhibitor. This case highlights the potential of JAK inhibitors in treating EBP, although long-term safety requires further investigation.

## Introduction

Epidermolysis bullosa (EB) encompasses a group of genetically determined skin disorders characterized by extreme skin fragility and the formation of blisters upon minor trauma (1). Based on the cleavage level at the dermal-epidermal junction, EB is classified into four main types: epidermolysis bullosa simplex, junctional EB, dystrophic EB (DEB), and Kindler syndrome (2, 3). DEB is characterized by recurrent episodes of pruritic blisters, which gradually progress to atrophy and scarring (4). Clinical onset may occur in adulthood and is frequently accompanied by nail dystrophy of varying severity (4). Dystrophic epidermolysis bullosa pruriginosa, also known as epidermolysis bullosa pruriginosa (EBP), is a rare clinical subtype of DEB. In addition to the common manifestations of skin blisters and ulcers, patients with EBP also present a unique feature, namely severe pruritus (5). The disease is inherited in an autosomal dominant or recessive pattern, and the causative gene is COL7A1, which encodes type VII collagen, a crucial component of anchoring fibrils at the dermal-epidermal junction (6). Diagnosis typically involves a combination of clinical presentation, histopathological examination, and comprehensive genetic assessment. However, traditional therapeutic approaches, including topical corticosteroids, tacrolimus, thalidomide, and systemic immunosuppressants, have shown limited effectiveness. Recently, biologic drugs and small-molecule inhibitors have expanded the treatment options for this disease (7, 8).

## Case presentation

A 59-year-old male presented to our department with nodules, scars, and severe pruritus on both lower extremities that had persisted for more than 30 years. The patient reported the onset of pruritus on both lower legs approximately 30 years earlier, with no identifiable cause; systemic conditions commonly associated with pruritus were excluded. After scratching, blisters formed and subsequently ruptured, gradually evolving into nodules and scars. Despite seeking medical advice at other hospitals, a definitive diagnosis was not established, and topical medications were ineffective. Over time, the number of skin lesions increased, and the pruritus became intense. There was no family history of similar skin diseases.

**Figure 1:**
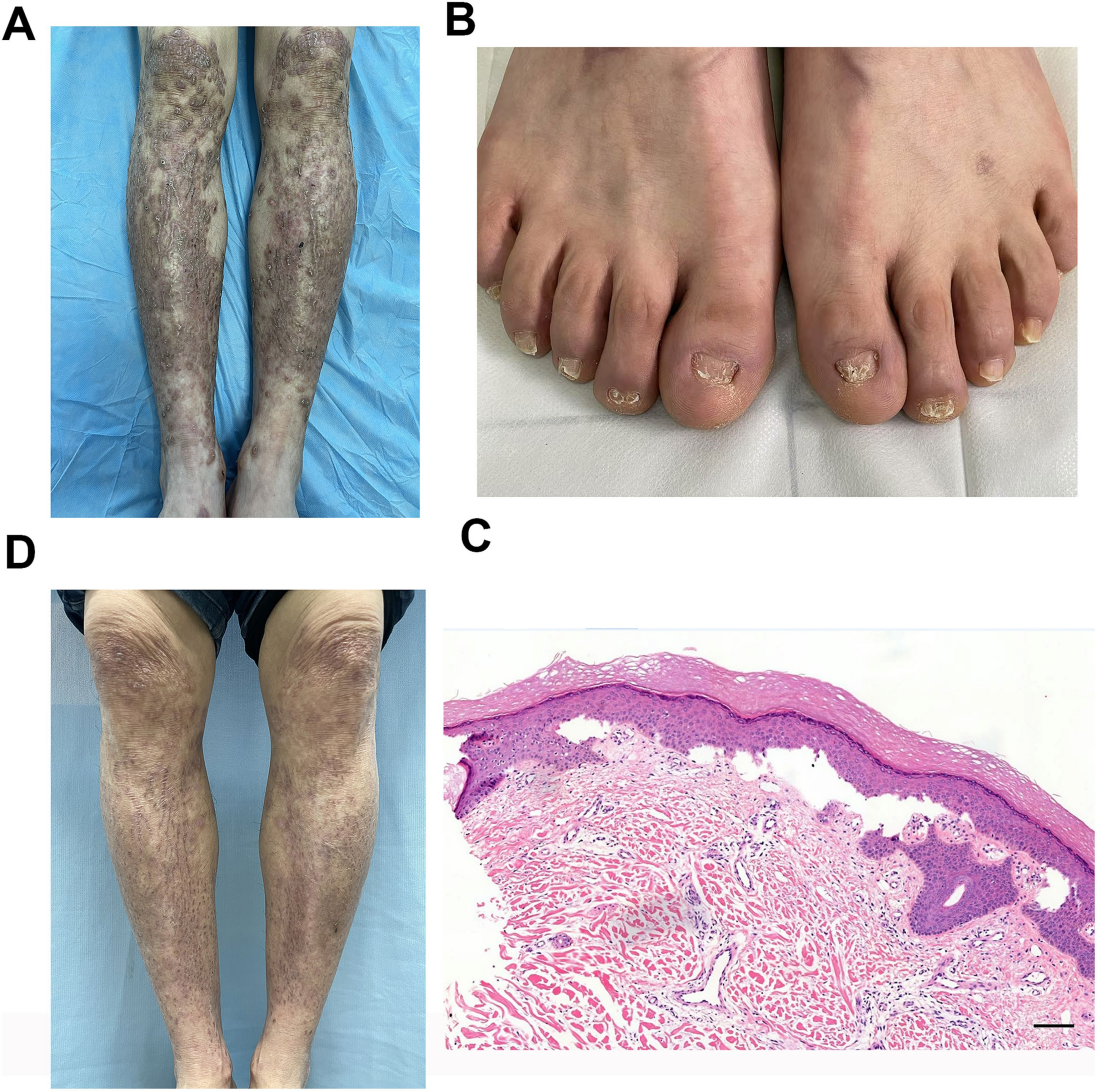
**(A)** Typical skin lesions of brownish-red nodules the size of soybeans and linear scars on the pretibial area. **(B)** Characteristic dystrophic nail changes included onychatrophy, onycholysis, onychorrhexis, and anonychia. **(C)** Histopathological examination of pretibial skin lesions revealed hyperkeratosis of the epidermis, thickening of the granular layer, mild acanthosis, dermo-epidermal cleavage, significant proliferation of small blood vessels in the dermal papillary layer, and a substantial infiltration of lymphocytes, plasma cells, and eosinophils around the vessels (H&E staining, scale bar = 100 μm). **(D)** Marked improvement of the pretibial area at the 10-month follow-up visit.

On physical examination, numerous brownish-red nodules the size of soybeans and linear scars were densely distributed on the extensor surfaces of both lower extremities. Some lesions had scales, scratch marks, and crusts, and a few dried blisters were also observed. Scattered brownish-red nodules were present on both thighs (Figure 1A). The toenails showed dystrophic changes such as onychatrophy, onycholysis, onychorrhexis, and even anonychia (Figure 1B). The visual analog scale (VAS) score for pruritus was 8/10.

Histopathological examination of a skin biopsy revealed hyperkeratosis of the epidermis, thickening of the granular layer, mild acanthosis, dermo-epidermal cleavage, significant proliferation of small blood vessels in the dermal papillary layer, and a substantial infiltration of lymphocytes, plasma cells, and eosinophils around the vessels (Figure 1C). Given the patient’s clinical manifestations, differential diagnoses including pemphigus and pemphigoid were initially considered. To rule out these autoimmune blistering disorders, serological testing for pathogenic antibodies was performed, which revealed negative results for desmoglein 1 (Dsg1), desmoglein 3 (Dsg3), bullous pemphigoid 180 (BP180), and bullous pemphigoid 230 (BP230). Thus, pemphigus and pemphigoid were excluded from the final diagnosis. Additionally, genetic testing revealed a heterozygous missense mutation in COL7A1: c.8201G>A (p.Gly2734Asp). Based on the clinical manifestations, immunological and histopathological findings, and genetic results, a diagnosis of EBP was made. The patient was prescribed tofacitinib at a dose of 5 mg orally twice daily with no topical medications administered. After 10 months of treatment, follow-up showed marked improvement in skin lesions (Figure 1D) and pruritus (VAS score: 1/10). Safety monitoring remained normal throughout therapy. Monitored items included clinical evaluation for infections (including screening for tuberculosis, hepatitis B, hepatitis C, HIV, and syphilis) and laboratory tests for liver function, renal function, complete blood count, lipid profile, and tumor markers.

## Discussion

In this study, we report a case of a patient with EBP harboring a heterozygous mutation in the COL7A1 gene, specifically c.8201G>A (p.Gly2734Asp). This mutation has been previously documented in the Polish DEB population (9) and is currently curated in the Human Gene Mutation Database (HGMD) (10). This variant probably falls into the category of autosomal dominantly inherited mutations. The proband’s father, a carrier of the c.8201G>A (p.Gly2734Asp) variant, displayed transient toenail loss, and this sign is potentially linked to EB (9). However, research on this specific mutation remains limited. Its impacts on the structure and function of the COL7A1 gene, which encodes the type VII collagen protein, have not yet been fully elucidated. The present study further supports that mutations at this locus can lead to EBP.

Pruritus is the most common and challenging symptom to manage in EBP patients. It initiates a vicious cycle of itching, scratching, and wound formation. Currently, the exact pathophysiology of pruritus in EB remains incompletely understood. The primary goals of treatment are to relieve pruritus and improve skin lesions. Several therapeutic approaches, including emollients, topical corticosteroids, oral antihistamines, gabapentin, thalidomide, and immunosuppressants, have been reported in the literature, but they often yield unsatisfactory results.

Some studies have reported successful treatment of DEB with dupilumab (7, 8), a human monoclonal antibody targeting interleukin (IL)-4 and IL-13, suggesting that DEB may be driven by Th2-mediated immune mechanisms (11–13). Another study examining the wound transcriptome profile of patients with EB revealed abnormally elevated expression of inflammation-related genes in EB lesions. These include IL-1A, IL-1B, IL-6, and IL-8, as well as components of the Janus kinase (JAK)-signal transducer and activator of transcription (STAT) pathway, compared with normal skin from the same patients and with healthy controls (14). In contrast, JAK inhibitors broadly suppress the JAK–STAT pathway, which is used by multiple cytokines, including IL-4, IL-13, and IL-31, thereby exerting a more comprehensive antipruritic effect (15). Tofacitinib, a pan-JAK inhibitor, primarily inhibits JAK1 and JAK3, with weaker inhibitory effects on JAK2 and TYK2 (16). In this study, tofacitinib demonstrated significant therapeutic efficacy in treating EBP. To the best of our knowledge, this is among the first reported cases of EBP treated with tofacitinib, further supporting its therapeutic potential (17, 18). Previous studies have also shown that oral JAK inhibitors (such as baricitinib and upadacitinib) may be more effective than dupilumab, but adverse reactions should be carefully monitored (8, 19). To place our case in the broader context of targeted therapies for DEB, we summarize the key characteristics of biologic drugs and JAK inhibitors reported for its management (Table 1).

**Table 1:** Biologic drugs and JAK inhibitors used in DEB.

Biologic drugs and JAK inhibitors in DEB		Mechanisms	Cases	References
Biologic drugs	Dupilumab	Inhibition of the Th2 pathway	37	(7, 8, 19)
JAK inhibitors	Baricitinib	Inhibition of JAK1 and JAK2	7	(20–22)
	Upadacitinib	Selective inhibition of JAK1	11	(21, 23–27)
	Abrocitinib	Selective inhibition of JAK1	1	(19)
	Tofacitinib	Inhibition of pan-JAK	2	(17, 18)

DEB, dystrophic epidermolysis bullosa; JAK, Janus kinase; STAT, signal transducer and activator of transcription.

## Conclusion

JAK inhibitors, such as tofacitinib, appear to be effective treatment options for EBP. Given the need for continuous treatment in EBP patients, the long-term safety of JAK inhibitors requires further investigation. Additional rigorous studies are needed to validate these efficacy observations and to develop comprehensive safety guidelines for the long-term use of JAK inhibitors in EBP patients.

## Data Availability

The original contributions presented in the study are included in the article/supplementary material, further inquiries can be directed to the corresponding author.
